# Editorial Comment: New Insights on the Mechanisms Affecting Fertility in Men with Non-Seminoma Testicular Cancer before Cancer Therapy

**DOI:** 10.1590/S1677-5538.IBJU.2020.02.07

**Published:** 2020-01-10

**Authors:** Gustavo Cardoso Guimarães

**Affiliations:** 1 Departamento de Oncologia Cirúrgica Beneficência Portuguesa de São Paulo São PauloSP Brasil Chefe do Departamento de Oncologia Cirúrgica, Beneficência Portuguesa de São Paulo, São Paulo, SP, Brasil

Dias TR ^1,2,3^, Agarwal A ^4^, Pushparaj PN ^5^, Ahmad G ^6^, Sharma R ^1^

^1^ American Center for Reproductive Medicine, Cleveland Clinic, Cleveland, OH, USA; ^2^ Universidade da Beira Interior, Covilhã, Portugal; ^3^ Department of Microscopy, Laboratory of Cell Biology, Institute of Biomedical Sciences Abel Salazar and Unit for Multidisciplinary Research in Biomedicine, University of Porto, Porto, Portugal; ^4^ American Center for Reproductive Medicine, Cleveland Clinic, Cleveland, OH, USA; ^5^ Center of Excellence in Genomic Medicine Research, Faculty of Applied Medical Sciences, Jeddah, Saudi Arabia; ^6^ Division of Pathology, School of Medical Sciences, Sydney University, Sydney, Australia

World J Mens Health. 2018 Dec 21. [Epub ahead of print]

**DOI:** 10.5534/wjmh.180099 | **ACCESS:** 10.5534/wjmh.180099

## COMMENT

In this paper, Tania Dias and colleagues, try to compare the sperm proteome of patients with NSTC, who cryopreserved their sperm before starting cancer treatment, with that from healthy fertile men.

Because patients with non-seminoma testicular cancer (NSTC) cancer can be subfertile or infertile, and present reduced sperm quality and the underlying mechanisms are unknown, this paper may help to identify the underlie causes involved with this condition.

The authors collected Semen samples after 2 to 3 days of abstinence and evaluated Volume, sperm motility, and sperm concentration according to World Health Organization (WHO) 2010 guidelines. Semen samples were then cryopreserved in TEST-yolk buffer and stored in liquid nitrogen at -196°C.

The identification of the differentially expressed proteins (DEPs) between the control and NSTC groups was con- ducted via Scaffold software. (Proteome Software Inc., Portland, OR, USA)

The criteria for the selection of DEPs for validation by Western blot (WB) included: 1) proteins involved in reproductive system development and function; 2) proteins involved in the top canonical pathways; 3) proteins with a higher difference of abundance between the experimental groups; 4) proteins with a well-described function in the literature

The authos identified 189 differentially expressed proteins (DEPs) in the dataset, from which five DEPs related to sperm function and fertilization were selected for validation by Western blot. And they indentified that underexpression of the mitochondrial complex subunits NADH:Ubiquinone Oxidoreductase Core Subunit S1 (NDUFS1) and ubiquinol-cytochrome C reductase core protein 2 (UQCRC2), as well as the underexpression of the testis-specific sodium/potassium-transporting ATPase subunit alpha-4 (ATP1A4) in the NSTC group.

The results indicate that sperm mitochondrial dysfunction may explain the observed decrease in sperm concentration, total sperm count and total motile count in NSTC patients. The identified DEPs may serve as potential biomarkers for the pathophysiology of subfertility/infertility in patients with NSTC.

